# Association between vitamin levels and geriatric hip fractures: A cross-sectional study

**DOI:** 10.3389/fnut.2025.1421257

**Published:** 2025-01-29

**Authors:** Qifei He, Chun Zhang, Wei Xie, Zhaoqiang Deng, Shiwei Yang, Xia Li, Wei Sun

**Affiliations:** ^1^Department of Orthopedics, Shenzhen Institute of Translational Medicine, The First Affiliated Hospital of Shenzhen University, Shenzhen Second People's Hospital, Shenzhen, Guangdong, China; ^2^School of Public Health and Emergency Management, Southern University of Science and Technology, Shenzhen, Guangdong, China

**Keywords:** nutrition, aging, vitamin, geriatric hip fracture, Retinol

## Abstract

**Objectives:**

Geriatric hip fractures, known as osteoporotic fractures, are associated with nutritional deficiencies such as vitamin D (VitD). However, the relationship between other vitamins besides VitD and fracture is still lacking. Therefore, we intended to identify various vitamin levels and deficiencies in elderly patients with hip fractures and compared those with healthy elderly individuals.

**Methods:**

A total of 210 geriatric hip fracture continuous patients and 402 healthy elderly individuals aged ≥65 years old and with complete serum vitamin records were enrolled in this study. The levels of serum VitA, VitB1, VitB2, VitB3, VitB5, VitB6, VitB9, VitE, VitK1, and 25-hydroxyvitamin D (25-HO-VitD) and nutritional markers, such as prognostic nutritional index (PNI), were compared between geriatric hip fracture patients and healthy control. The correlation between vitamin level and the nutritional markers was explored. A multiple linear regression analysis was conducted to assess the association between vitamin levels and hip fracture.

**Results:**

The levels of VitA, VitB1, VitB3, VitB5, VitB9, 25-HO-VitD, 25-HO-VitD3, E, and K1 were all lower in the hip fracture group (*p* < 0.05). More than 80% of older persons suffered 25-HO-VitD deficiency in both healthy and fracture groups. A positive correlation between levels of VitA and nutritional markers existed in the fracture and healthy groups. In regression analysis, the association strength between standardized vitamin levels and fracture was the most significant in VitB9 (*β* = −0.94; 95%CI, −1.15 to −0.73; *p* < 0.001), VitA (*β* = −0.83; 95%CI, −1.04 to −0.61; *p* < 0.001), and VitK1 (*β* = −0.80; 95%CI, −1.02 to −0.58; *p* < 0.001), with no significant statistic difference found in VitB2, VitB9, 25-HO-VitD2, and 25-HO-VitD3.

**Conclusion:**

Vitamin D deficiency is common in elderly people with or without fracture. The levels of VitA, VitB9, and VitK1, instead of VitD, were much lower in fracture patients than in the healthy control, even controlling age and gender. VitA is a potential target for hip fracture prevention.

## Introduction

With worldwide growth in aging populations, an increasing number of people suffer from osteoporotic fractures, especially hip fractures. Hip fracture becomes a severe public health concern because of the associated high risk of morbidity (1), mortality (2), and disability (3), as well as high societal healthcare costs (1). At present, known factors associated with the incidence of fractures include physical age (4), vitamin D and calcium supplements (5), and nutritional intake (6–8). Increasing evidence indicates that a variety of micronutrients, apart from Ca^2+^ and vitamin D, such as minerals and trace elements, vitamins, and polyphenols can affect the development of osteoporosis (9, 10). Studies have shown that vitamins A, E, K, and B play a role in regulating bone metabolism and that deficiencies in these vitamins could be considered dietary risk factors for osteoporosis (11). However, the understanding of the associations between various vitamin levels and geriatric osteoporotic hip fractures has yet to be clearly defined.

Vitamins are essential nutrients for maintaining health. Vitamin D is closely related to osteoporosis (9, 10), and its deficiency increases the risk of hip fractures (12). In addition, vitamin K intake promotes bone health and reduces the risk of fractures (13, 14). B vitamins are involved in homocysteine metabolism, which affects bone density and hip fracture. Deficiency in B vitamins may impair the capacity of balance in older adults, thereby increasing the risk of falls (15). Similarly, research has found a negative correlation between levels of vitamins C and E, and male hip fracture risk (16, 17). Although many studies have extensively investigated the relationship between vitamins and hip fracture risk and bone density, to date, no study has simultaneously investigated the deficiency of multiple vitamins in patients with hip fractures, nor has any study compared the relative risks of different vitamin deficiencies on fracture risk. Furthermore, the potential role of vitamins as a monitoring tool for the nutritional status of older patients remains unclear and requires further investigation.

Therefore, we conducted a cross-sectional study comparing the serum levels of vitamin A, B1, B2, B3, B5, B6, B9, E, K1, and 25-hydroxy vitamin D (25-OH-VitD) and vitamin deficiencies between geriatric hip fracture patients and healthy older individuals who underwent medical examinations during the same period. We also explored the correlation between vitamin levels and other nutritional indicators, and compared the standardized vitamin levels and their association with fractures after controlling for relevant factors such as age.

## Methods

### Study design and study population

**Figure 1:**
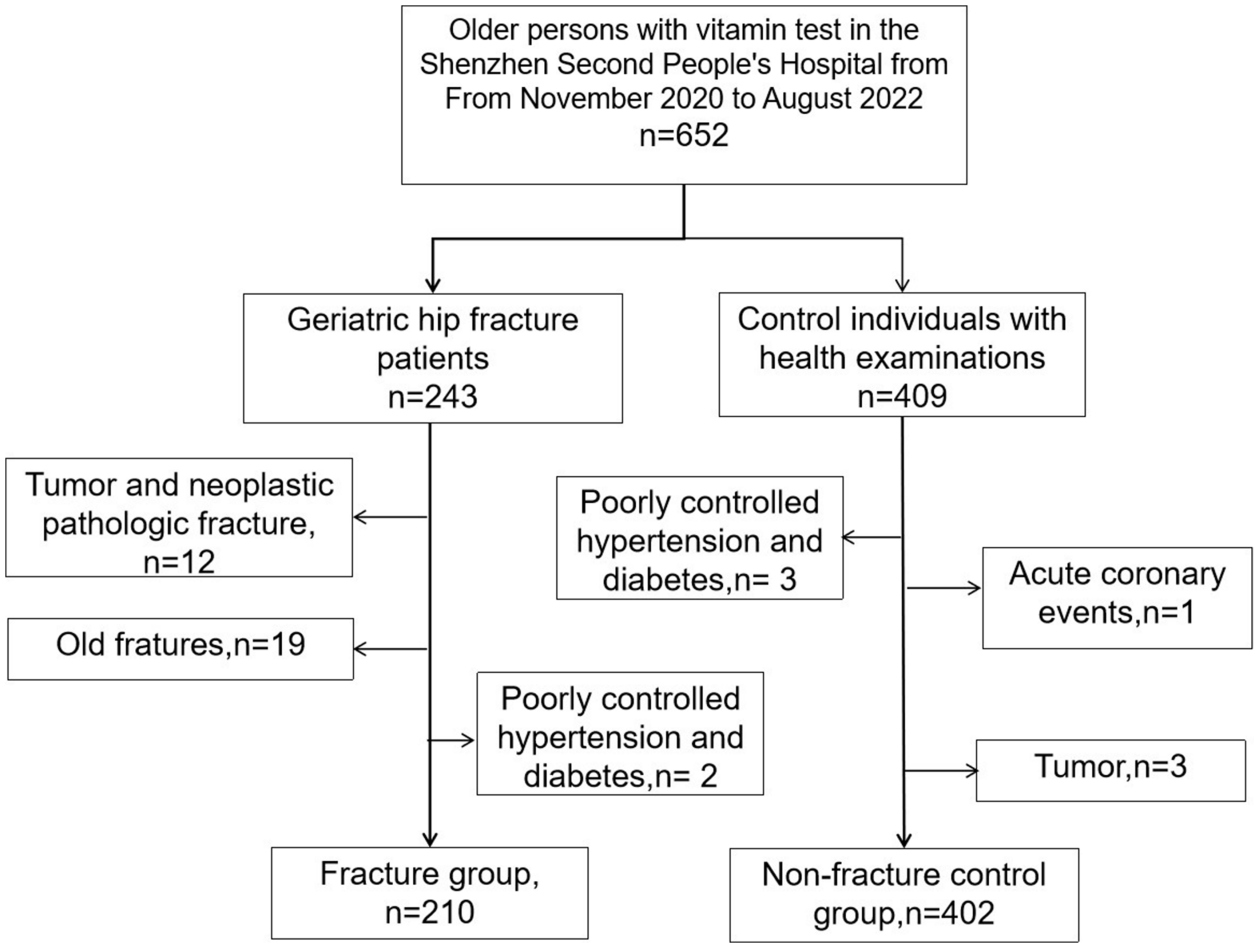
Flowchart of the patient enrollment process.

This cross-sectional study was approved by the ethics committee of Shenzhen Second People’s Hospital and was conducted following the principles of the Declaration of Helsinki. Participants were consecutive geriatric hip fracture patients and health examination older individuals over 65 years old, who underwent health examinations (including vitamin tests) and signed informed consent in the Shenzhen Second People’s Hospital (China) from November 2020 to August 2022 (Figure 1). The geriatric hip fracture patients were deemed the observation group, while the people undergoing health examination were the control group. The inclusion criteria of the observation group involved patients who: (I) were 65 years old and above, regardless of sex; (II) had completed the standardized examination and vitamin levels tests; (III) had been diagnosed as new onset hip fracture, which refers to the fractures less than 3 weeks. The inclusion criteria of the control group were the same as the observation group expect for the third piece.

Participants were excluded if they met one of the following criteria: (I) missing clinical data or lost follow-up; (II) have suffered tumor, poorly controlled hypertension and diabetes, acute coronary events, and other diseases that require hospitalization; (III) were unable to cooperate with the inspection for various reasons. According to the inclusion and exclusion criteria, 210 geriatric hip fracture patients were included in the observation group, while 402 healthy people were in the control group.

### Data collection

The demographic and laboratory data for age, gender, and vitamin levels were extracted and recorded from the hospital HIS system. Two doctors then reviewed the data for a second time. Vitamin laboratory data were obtained by collecting 2 mL of fasting blood from patients before surgery upon admission or during the medical examination. The content of vitamins in serum was measured using liquid chromatography–tandem mass spectrometry (LC–MS/MS) technique. The instrument used was the Xevo-TQD IVD liquid chromatography-tandem mass spectrometer from Waters Corporation in the United States, and the vitamin sample release reagent kit was provided by Hangzhou Bio-chinchem Co., Ltd. Each vitamin was divided into deficient and non-deficient groups based on whether its level was below the reference range (the reference range for each vitamin can be found in Supplementary Table S1). There is currently no unified normal reference value for 25-hydroxyvitamin D2 (25-OH-VitD2) and 25-hydroxyvitamin D3 (25-OH-VitD3), and their deficiency status was not determined.

The nutritional markers, including body mass index (BMI), serum albumin (Alb), hemoglobin (Hb), lymphocyte (Lym), and prognostic nutritional index (PNI) (18), were also retrieved from our HIS system. The marker PNI was calculated as described previously: PNI = Alb (g/L) + 5 × Lym (10^9/L).

### Statistics

Categorical data were presented as the counts (percentage); continuous data were presented as the mean (± standard deviation) if normally distributed or as the median (interquartile range) if not normally distributed. Baseline characteristics and vitamin levels between the fracture and control groups were first analyzed. Differences in continuous data, such as levels of vitamins and age, between the observation group and control group were analyzed by independent samples *T*-tests or median tests, depending on the normality of the data. The comparison of categorical variables such as sex and vitamin deficiency status between the two groups was performed using the chi-square test or Fisher’s exact test. Next, Pearson’s correlation was used to determine the correlation between vitamin level and the nutritional markers. A heatmap was used to illustrate their correlations. Finally, a multiple linear regression analysis was conducted to explore whether vitamin levels are different between the two groups after controlling age, gender, and BMI. To facilitate the comparison of the degree of difference between various vitamins and fractures, we standardized the vitamin levels. As the prevalence of osteoporosis and osteoporotic fracture are different among men and women (1, 18), we repeated the analysis separately for the men and women subgroup in the subgroup analysis and estimated *p*-values for interaction by introducing multiplicative terms between sex and vitamin to the regression models. All statistical tests were two-sided, and a *p*-value less than or equal to 0.05 was considered statistically significant. Statistical analysis was performed using the R 4.0 software.

## Results

### Clinical characteristics and vitamin levels between fracture and control groups

Table 1 depicts the clinical characteristics and vitamin levels of the fracture and non-fracture control groups. The median age of the fracture group [80.34 (65–97)] was observed to be higher compared to that of the control group [72.11 (65–90)] (*p* < 0.001). The BMI was significantly different between the fracture and non-fracture groups, with the fracture group showing a mean BMI of 22.1 ± 3.8, while the non-fracture group had a mean BMI of 25.0 ± 3.5 (*p* < 0.001). Notably, there was no significant difference in gender between the fracture and non-fracture groups (*p* = 0.547). The levels of serum 25-hydroxyvitamin D, D3, and vitamins A, B1, B3, B5, B9, E, and K1 were significantly lower in the fracture group as compared to the non-fracture group (all *p* < 0.05). Regarding the nutritional markers, patients in the fracture group demonstrated lower values of albumin (*p* < 0.001), hemoglobin (*p* < 0.001), lymphocyte (*p* < 0.001), and PNI (*p* < 0.001). The distribution plots of original vitamin levels in the Supplemental files, of which the majority of vitamin demonstrated skewed distribution (Supplementary Figure S1).

**Table 1:** Characteristics and vitamin levels between fracture and control groups.

Clinical data	Fracture group (*N* = 210)	Non-fracture control group (*N* = 402)	*p*-value
Age [years]	80.34 (8.14)	72.11 (5.91)	<0.001
Sex (*n*[%])			0.547
Man	62 (29.5)	108 (26.9)	
Woman	148 (70.5)	294 (73.1)	
BMI (kg/m^2^)	22.1 (3.8)	25.0 (3.5)	<0.001
Vitamin levels [ng/ml]			
25-HO-VitD (median [IQR])	17.30 [12.75, 24.21]	21.51 [16.63, 27.96]	<0.001
25-HO-VitD2 (median [IQR])	1.07 [0.72, 1.58]	1.05 [0.64, 1.77]	0.991
25-HO-VitD3 (median [IQR])	17.57 [11.38, 24.54]	19.96 [15.10, 26.12]	0.004
VitA (median [IQR])	282.62 [190.95, 384.37]	433.10 [346.99, 546.60]	<0.001
VitB1 (median [IQR])	1.97 [1.46, 2.93]	2.68 [2.06, 3.84]	<0.001
VitB2 (median [IQR])	8.65 [5.65, 14.92]	9.78 [6.77, 15.99]	0.168
VitB3 (median [IQR])	10.35 [6.75, 18.55]	21.30 [13.07, 28.96]	<0.001
VitB5 (median [IQR])	50.82 [39.33, 67.38]	56.08 [44.72, 74.47]	0.034
VitB6 (median [IQR])	4.29 [2.52, 8.87]	5.23 [3.15, 8.73]	0.115
VitB9 (median [IQR])	3.59 [2.61, 7.56]	10.40 [5.64, 17.72]	<0.001
VitE (median [IQR])	7.69 [5.85, 9.82]	9.03 [7.36, 11.16]	<0.001
VitK1 (median [IQR])	0.42 [0.26, 0.72]	1.12 [0.69, 1.68]	<0.001
Nutritional markers			
Alb (median [IQR])	37.70 [34.30, 40.30]	41.30 [39.00, 43.70]	<0.001
Hb [mean (SD)]	113.00 [100.75, 125.25]	127.00 [118.00, 135.00]	<0.001
Lymphocyte (median [IQR])	1.11 [0.77, 1.49]	1.85 [1.49, 2.28]	<0.001
PNI [mean (SD)]	43.16 (6.27)	50.98 (5.73)	<0.001

25-OH-VitD, 25-hydroxy vitamin D; 25-OH-VitD2, 25-hydroxyvitamin D2; 25-OH-VitD3, 25-hydroxyvitamin D3; VitA, vitamin A; VitB1, vitamin B1; VitB2, vitamin B2; VitB3, vitamin B3; VitB5, vitamin B5; VitB6, vitamin B6; VitB9, vitamin B9; VitE, vitamin E; VitK1, vitamin K1; Alb, albumin; BMI, body mass index; Hb, hemoglobin; PNI, prognostic nutritional index.

### Vitamin deficient status between fracture and control group

While the fracture group exhibited lower vitamin levels, it does not mean it is a deficiency in vitamins that needs to be treated in this group. Therefore, we conducted a comparison of vitamin deficiencies between the fracture and control groups. Among older patients with hip fractures, 60.0% were deficient in vitamin A, 61.3% in vitamin B1, 23% in vitamin B3, and 55.7% in vitamin B9 (Table 2). Furthermore, statistically significant differences were observed between the fracture and non-fracture groups regarding age and deficiencies in vitamins A, B1, B3, B9, and E (*p* < 0.05). Although both the fracture group (85.6%) and the control group (81.1%) commonly exhibited a deficiency in 25-hydroxyvitamin D, the proportion of 25-hydroxyvitamin D between fracture and non-fracture patients did not demonstrate statistical significance (*p* = 0.218).

**Table 2:** Vitamin deficiency rate between fracture and control groups.

Vitamin type*	Fracture Group 210 (%)	Non-fracture control group 402 (%)	*P*-value
25-HO-VitD	166 (85.6)	308 (81.1)	0.218
VitA	75 (60.0)	57 (19.5)	<0.001
VitB1	76 (61.3)	113 (38.7)	<0.001
VitB2	2 (1.6)	2 (0.7)	0.741
VitB3	23 (18.5)	14 (4.8)	<0.001
VitB5	0 (0.0)	1 (0.3)	0.251
VitB6	70 (56.0)	137 (46.9)	0.111
VitB9	78 (55.7)	54 (17.6)	<0.001
VitE	1 (0.8)	5 (1.7)	0.006
VitK1	2 (1.6)	1 (0.3)	0.449

*As there is no uniform normal reference value for 25-OH-VitD2 and 25-OH-VitD3, the deficiency of these two variables was not included.

25-OH-VitD, 25-hydroxy vitamin D; VitA, vitamin A; VitB1, vitamin B1; VitB2, vitamin B2; VitB3, vitamin B3; VitB5, vitamin B5; VitB6, vitamin B6; VitB9, vitamin B9; VitE, vitamin E; VitK1, vitamin K1.

### Associations between standardized vitamin levels and hip fractures in observational and control groups

To investigate whether vitamin levels are different between fractures and non-fractures, univariate and multivariate linear regression analyses were performed (Table 3). In univariate analysis, standardized levels of vitamins except for 25-HO-VitD2, 25-HO-VitD3, VitB2, and VitB3 were significantly associated with fracture. The distribution plots of original and standardized vitamin levels were presented in Supplementary Figures S1, S2. After controlling for age, gender, and BMI, multivariate linear regression analysis revealed that significant differences in standardized vitamin levels remained between the fracture and non-fracture groups for vitamins B9, A, K1, B3, B1, B5, E, as well as 25-OH-VitD. Among these, the disparities of vitamin B9 (*β* = −0.94; 95%CI, −1.15 to −0.73; *p* < 0.001) between the groups were found to be the most significant, followed by vitamin A (*β* = −0.77; 95%CI, −0.99 to −0.55; *p* < 0.001), K1 (*β* = −0.77; 95%CI, −1.00 to −0.54; *p* < 0.001), B3 (*β* = −0.59; 95%CI, −0.75 to −0.25; *p* < 0.001), B1 (*β* = −0.61; 95%CI, −0.86 to −0.37; *p* < 0.001), B5 (*β* = −0.50; 95%CI, −0.75 to −0.25; *p* < 0.001), E (*β* = −0.40; 95%CI, −0.64 to −0.16; *p* = 0.001), as well as 25-OH vitamin D (*β* = −0.30; 95%CI, −0.50 to −0.10; *p* = 0.004). However, no significant differences were observed between the two groups regarding 25-OH-VitD2 (*β* = −0.04; 95%CI, −0.29 to 0.22; *p* = 0.768), 25-OH-VitD3 (*β* = −0.25; 95%CI, −0.48 to 0.01; *p* = 0.066), as well as vitamins B2 (*β* = −0.28; 95%CI, −0.54 to −0.03; *p* = 0.028) and B6 (*β* = −0.25; 95%CI, −0.50 to 0.01; *p* = 0.012). In the sex-stratified subgroup analysis, we found that the discrepancy in vitamins D, A, K1, B1, B3, B5, B9, and E was significant between fracture and non-fracture groups, regardless of gender difference (Supplementary Table S2). This finding is consistent with the result drawn from the overall population. In addition, we found that the associations between vitamin B1 and fracture were significantly different across the sexes (*P* for interaction = 0.001), with the effect size being relatively stronger among men.

**Table 3:** Univariate and multivariate regression analysis of standardized vitamin levels between fracture and control group.

Item	Univariate linear regression analysis		Multivariate linear regression analysis	
	*β*(95% CI)	*P*-value	*β*(95% CI)	*P*-value
25-HO-VitD_inorm	−0.44(−0.61,−0.27)	<0.001	−0.30(−0.50,−0.10)	0.004
25-HO-VitD2_inorm	0.00(−0.21,0.21)	0.985	−0.04(−0.29,0.22)	0.768
25-HO-VitD3_inorm	−0.33(−0.54,−0.12)	0.002	−0.23(−0.48,0.01)	0.066
VitA_inorm	−0.97(−1.16,−0.78)	<0.001	−0.77(−0.99,−0.55)	<0.001
VitB1_inorm	−0.57(−0.78,−0.37)	<0.001	−0.61(−0.86,−0.37)	<0.001
VitB2_inorm	−0.12(−0.33,0.09)	0.252	−0.28(−0.54,−0.03)	0.028
VitB3_inorm	−0.72(−0.92,−0.52)	<0.001	−0.59(−0.83,−0.35)	<0.001
VitB5_inorm	−0.24(−0.45,−0.03)	0.024	−0.50(−0.75,−0.25)	<0.001
VitB6_inorm	−0.16(−0.37,0.05)	0.139	−0.24(−0.50,0.01)	0.058
VitB9_inorm	−0.84(−1.03,−0.66)	<0.001	−0.97(−1.19,−0.75)	<0.001
VitE_inorm	−0.45(−0.65,−0.24)	<0.001	−0.40(−0.64,−0.16)	0.001
VitK1_inorm	−0.94(−1.13,−0.76)	<0.001	−0.77(−1.00,−0.54)	<0.001

95%CI, 95% confidence interval; 25-OH-VitD, 25-hydroxy vitamin D; 25-OH-VitD2, 25-hydroxyvitamin D2; 25-OH-VitD3, 25-hydroxyvitamin D3; VitA, vitamin A; VitB1, vitamin B1; VitB2, vitamin B2; VitB3, vitamin B3; VitB5, vitamin B5; VitB6, vitamin B6; VitB9, vitamin B9; VitE, vitamin E; VitK1, vitamin K1; Alb, albumin; Hb, hemoglobin; PNI, prognostic nutritional index.

### Correlation between vitamin levels and the nutritional markers

**Figure 2:**
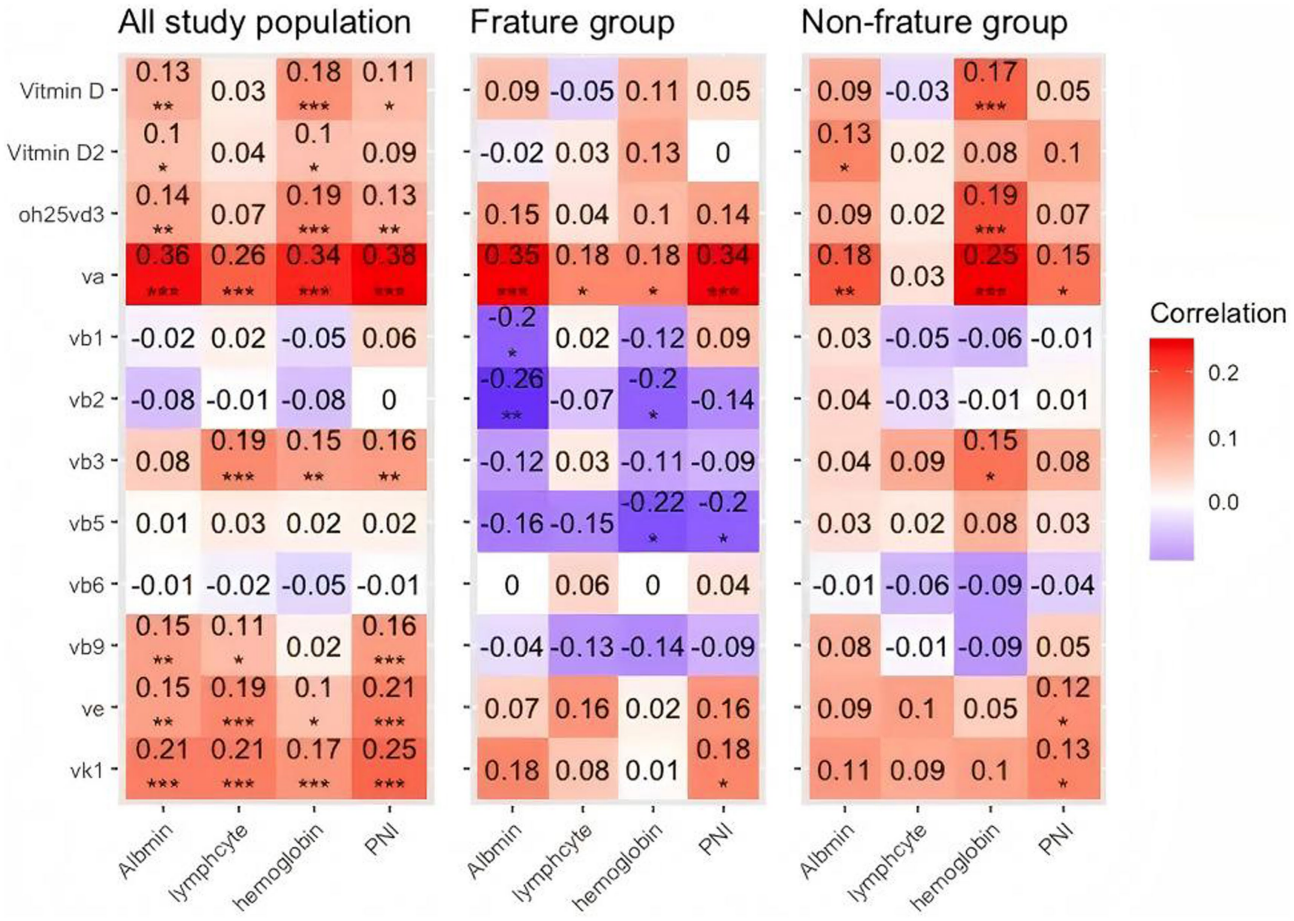
Heatmap displaying the correlation matrix of various vitamins and nutritional markers. Each cell represents the Pearson correlation coefficient between pairs of variables, with color intensity indicating the strength of the correlation. Red indicates a positive correlation, while blue indicates a negative correlation. Correlation values range from −1 to +1, with values closer to 1 or −1 denoting stronger relationships. *p*-values are indicated with asterisks as follows: *p < 0.05, **p < 0.01, ***p < 0.001, highlighting the statistical significance of the correlations.

Correlation analysis results between vitamin levels and the nutritional markers are shown in Figure 2. There was a positive correlation between levels of vitamin A and albumin (*r* = 0.36; *p* < 0.001), lymphocyte (*r* = 0.26; *p* < 0.001), hemoglobin (*r* = 0.34; *p* < 0.001), and PNI (*r* = 0.38; *p* < 0.001) in the whole patients. Similarly, in the fracture group, the positive correlation between vitamin A and albumin (*r* = 0.35; *p* < 0.001), lymphocyte (*r* = 0.18; *p* < 0.05), hemoglobin (*r* = 0.18; *p* < 0.05), and PNI (*r* = 0.34; *p* < 0.001) were also found. The relationship existed in the control group. The correlation coefficient of the control group between vitamin A and albumin, vitamin A and lymphocyte, and vitamin A and PNI were 0.18 (*p* < 0.01), 0.25 (*p* < 0.001), and 0.15 (*p* < 0.15), respectively. Vitamin A potentially serves as a nutritional marker. The heatmap of correlations between vitamin levels within the three groups (all study populations, fracture group, and non-fracture group) was shown in Supplementary Figure S3.

## Discussion

This cross-sectional study examined the relationship between hip fracture and vitamins. We found that the fracture group displayed lower levels of vitamins and nutritional markers compared to the non-fracture group. Both fracture and healthy geriatric people suffered the vitamin D deficiency. Serum vitamins B9, A, K1, B3, B1, B5, and E, aside from 25-hydroxyvitamin D, exhibited differences between the fracture and non-fracture groups, especially with the largest differences in vitamin B9, A, and K1. Besides, a positive correlation between vitamin A levels and nutritional markers was observed in geriatric populations.

In this study, vitamin D deficiency was prevalent among older patients, regardless of whether they had fractures or not. Over 80% of older patients exhibited insufficient levels of vitamin D. Niafar et al. (19) found that approximately 82% of postmenopausal women suffered from a deficiency in 25-hydroxy vitamin D. Similarly, Mateopascual et al. (20) discovered that 86.3% of community-dwelling individuals aged over 65 years suffered from vitamin D deficiency. These findings align closely with our results that vitamin D deficiency is widespread in the older. Consequently, it suggests that screening and assessment of vitamin D deficiency should be conducted as part of routine care for all older individuals.

Apart from vitamin D, our study found vitamins A and K also exhibited significant differences between older patients with hip fractures and healthy controls. The relationship between vitamin A and the risk of osteoporotic fractures is controversial. Within the cohort of elderly participants in the Norwegian Epidemiologic Osteoporosis Studies (NOREPOS), no association was detected between elevated serum vitamin A levels and an increased risk for fractures (21). Furthermore, a comprehensive meta-analysis of the available data has revealed that augmented intake of vitamin A does not confer an increased risk of fractures (22). Whereas, a study, based on the European Prospective Investigation into Cancer and Nutrition (EPIC)-Norfolk cohort, found that higher plasma concentrations of *α*-carotene and *β*-carotene were linked to a reduced risk of hip fractures in men (23). The inconsistency in these findings highlights the complexity of vitamin A’s role in bone health and the need for further research to elucidate the mechanisms underlying its effects on bone metabolism and fracture susceptibility.

As mentioned above, we observed that elderly patients exhibited deficiencies in vitamin D, both with and without fractures, while the difference in vitamin A levels between the fractured and non-fractured elderly was more pronounced. This finding aligns with the literature, which suggests that the direct relationship between vitamin A and poor bone health was more pronounced in individuals with obesity or vitamin D deficiency (24). Our results might support the hypothesis that vitamin A and vitamin D may interact in a way that vitamin A could exacerbate the risk of fractures in patients with vitamin D deficiency. These results indicated that to improve bone health, vitamin A could be monitored among the elderly population, especially those with vitamin D deficiency.

Besides vitamin A, vitamin K is associated with osteoporosis (25). Vitamin K serves as an important cofactor in the carboxylation of many bone-forming proteins (26). Vitamin K deficiency can lead to undercarboxylation of osteocalcin, and the combination of vitamin K deficiency and undercarboxylated osteocalcin can lower bone density and increase the risk of osteoporosis (27). Population-based studies also suggest that dietary supplementation of vitamin K can reduce bone loss in postmenopausal women by approximately 35% compared to a placebo (12). The level of vitamin K is also related to the risk of fractures in postmenopausal women. For every 1 μg/L rise in serum vitamin K1 (phylloquinone) concentrations, there is a 45% decrease in the risk of fractures among post-menopausal women with osteoporosis as the result of enhanced hip strength (28). Another meta-analysis reported a positive influence of vitamin K on BMD and fracture risk (29). In this study, we also found a strong association between vitamin K and hip fracture. A previous meta-analysis demonstrated the combination effect of vitamin K and D on increasing total BMD (30). The role of a combination of vitamin K and D in reducing osteoporotic fracture risk merits further exploration.

The role of vitamin B9 (serum folic acid) on BMD has been investigated in several studies. Circulating plasma vitamin B9 concentrations were associated with BMD and bone biomechanical strength in post-menopausal Chinese-Singaporean women and those who underwent hip arthroplasty (31). A combination of vitamin B9 and D intake significantly improved overall cognitive function and attention (TMTB) compared to the placebo group in patients with mild cognitive impairment and animal models of Alzheimer’s disease (32, 33). These data suggested that patients with a deficiency of both vitamin B9 and D might suffer a high fall risk as a result of cognitive impairment. Taken together, supplementing vitamins A, B9, and K, besides vitamin D in older individuals may play an additional role in reducing the risk of osteoporotic hip fractures, but further prospective cohort studies are needed to confirm the result.

In this study, we found an interesting positive correlation between vitamin A levels and nutritional markers. Vitamin A deficiency indicates overall nutritional deficiency, which may hinder bone growth, increasing the occurrence of hip fracture. Moreover, Vitamin A deficiency can impair the body’s ability to absorb and utilize other nutrients effectively (34). For instance, it can affect the absorption of fat-soluble vitamins (such as vitamins D, E, and K) and limit the conversion of provitamin A carotenoids found in fruits and vegetables into active vitamin A. Besides, Vitamin A plays a crucial role in maintaining a healthy immune system. Its deficiency can weaken the immune response, making individuals more susceptible to infections and illnesses, which can further compromise overall nutritional status by affecting appetite, nutrient absorption, and nutrient utilization (35). Vitamin A deficiency can have progressive, negative effects on vital processes of the human body. Vitamin A deficiency not only affects bone metabolism but also indicates overall nutritional deficiency. However, the correlation between vitamin A and nutritional markers was not very strong. Weak to moderate correlations were observed in a previous study, which explored the correlations between free 25-HO-VitD and creatinine in the elderly population and suggested that vitamins, as a kind of nutrient, can only reflect part of the nutritional state (36). Interestingly, some correlations between vitamin levels and nutritional markers in the fracture were negative, for instance, Vitamin B2 and Albumin in the fracture group. The underlying mechanisms might be explained by increased oxidative stress in the patient group. Previous studies showed that increased oxidative stress status could be inversely correlated with riboflavin (vitamin B2) (37, 38) while positively related to oxidized albumin, especially among patients (39). More studies are needed to explore the role of vitamins, especially vitamins A and B2, in the whole body in different populations.

This study had some limitations. First, this study is a cross-sectional study, and thus, causal relationships between variables cannot be clarified. Longitudinal data are needed to enable better causal inferences. Moreover, this study does not control all confounding factors affecting geriatric hip fracture and vitamin levels. We did not explore the potential impact of bone mineral density (BMD) and history of falls, as the data were not recorded during baseline data collection. Further research is needed to investigate their influence.

In this study, we first comprehensively examine the levels of various vitamins in geriatric hip fractures. Moreover, we compare the levels of difference across varying vitamins between fracture and non-fracture patients through the standardization of vitamin levels. The strength of this study is in its identification of vitamins A, K1, and B9 as important markers, contributing to expanding our understanding of nutrition and bone health in elderly populations.

## Conclusion

The levels of various vitamins in geriatric hip fracture patients were lower than those of the healthy control people. Levels of vitamins A, K, B, and E in fracture patients were much lower than those without fracture, even controlling age and gender. Vitamin A is a potential nutritional marker and target for hip fracture prevention and treatment.

## Data Availability

The original contributions presented in the study are included in the article/Supplementary material, further inquiries can be directed to the corresponding authors.
