# Corrigendum: Association between vitamin levels and geriatric hip fractures: a cross-sectional study

**DOI:** 10.3389/fnut.2025.1595419

**Published:** 2025-04-01

**Authors:** Qifei He, Chun Zhang, Wei Xie, Zhaoqiang Deng, Shiwei Yang, Xia Li, Wei Sun

**Affiliations:** ^1^Department of Orthopedics, Shenzhen Institute of Translational Medicine, The First Affiliated Hospital of Shenzhen University, Shenzhen Second People's Hospital, Shenzhen, Guangdong, China; ^2^School of Public Health and Emergency Management, Southern University of Science and Technology, Shenzhen, Guangdong, China

**Keywords:** nutrition, aging, vitamin, geriatric hip fracture, Retinol

In the published article, there was an error in the **Funding statement**. The funder, Shenzhen Science and Technology Innovation Commission Sustainable Development Special Project, was not included. The statement read as follows:

“This study was supported by Postdoctoral Research Subsidy of Settling in Shenzhen, (No. 1040012); Shenzhen Second People's Hospital Key Clinical Research Fund of Shenzhen High-level Hospital Construction Project (No. 2022015); Shenzhen Second People's Hospital Clinical Research Fund of Guangdong Province High-level Hospital Construction Project (Nos. 20223357010, 20203357022, and 20213357004).”

The correct Funding statement appears below.

