# Supplementary material for: Association between vitamin levels and geriatric hip fractures: A cross-sectional study
Source: Front Nutr. 2025 Jan 29;12:1421257. doi: 10.3389/fnut.2025.1421257 (PMC11813756; doi:10.3389/fnut.2025.1421257)
Supplement: Supplementary file 1 [file Data_Sheet_1.doc]

Supplementary Table 1. The range for each vitamin.

| Vitamin type* | range | units |
| --- | --- | --- |
| 25-HO-VitD | 30.01-100.00 | ng/ml |
| VitA | 325.00-780.00 | ng/ml |
| VitB | 2.40-9.02 | ng/ml |
| VitB2 | 2.33-14.69 | ng/ml |
| VitB3 | 5.20-72.10 | ng/ml |
| VitB5 | 12.90-253.10 | ng/ml |
| VitB6 | 4.90-30.90 | ng/ml |
| VitB9 | >4.00 | ng/ml |
| VitE | 5.00-18.00 | ng/ml |
| VitK1 | 0.13-1.88 | ng/ml |

*The range of 25-OH-VitD2 and 25-OH-VitD3 was not provided by the measurement corporation.

Supplementary Table 2. Multivariate regression analysis of standardized vitamin levels between fracture and control group among men and women

| Item | Men | | Women | | P for interaction |
| --- | --- | --- | --- | --- | --- |
|  | β(95% CI) | P value | β(95% CI) | P value |  |
| 25-HO-VitD_inorm | -0.42(-0.80,-0.03) | 0.034 | -0.25(-0.48,-0.01) | 0.042 | 0.869 |
| 25-HO-VitD2_inorm | 0.18(-0.27,0.62) | 0.439 | -0.13(-0.44,0.18) | 0.399 | 0.263 |
| 25-HO-VitD3_inorm | -0.40(-0.85,0.06) | 0.090 | -0.16(-0.45,0.14) | 0.299 | 0.356 |
| VitA_inorm | -0.94(-1.33,-0.56) | <0.001 | -0.69(-0.96,-0.42) | <0.001 | 0.119 |
| VitB1_inorm | -1.02(-1.46,-0.57) | <0.001 | -0.44(-0.72,-0.16) | 0.002 | 0.001 |
| VitB2_inorm | -0.05(-0.54,0.44) | 0.842 | -0.38(-0.68,-0.09) | 0.011 | 0.275 |
| VitB3_inorm | -0.61(-1.06,-0.15) | 0.010 | -0.58(-0.86,-0.29) | <0.001 | 0.680 |
| VitB5_inorm | -0.55(-0.99,-0.10) | 0.017 | -0.47(-0.77,-0.17) | 0.003 | 0.269 |
| VitB6_inorm | -0.22(-0.72,0.27) | 0.382 | -0.25(-0.54,0.04) | 0.098 | 0.510 |
| VitB9_inorm | -0.71(-1.13,-0.29) | 0.001 | -1.09(-1.35,-0.83) | <0.001 | 0.370 |
| VitE_inorm | -0.44(-0.86,-0.03) | 0.038 | -0.38(-0.68,-0.09) | 0.011 | 0.666 |
| VitK1_inorm | -0.96(-1.36,-0.57) | <0.001 | -0.69(-0.97,-0.40) | <0.001 | 0.186 |

95%CI: 95% conﬁdence interval, 25-OH-VitD: 25-hydroxy vitamin D, 25-OH-VitD2: 25-hydroxyvitamin D2, 25-OH-VitD3: 25-hydroxyvitamin D3, VitA: vitamin A, VitB1: vitamin B1, VitB2: vitamin B2, VitB3: vitamin B3, VitB5: vitamin B5, VitB6: vitamin B6, VitB9: vitamin B9, VitE: vitamin E, VitK1: vitamin K1, Alb: albumin, Hb: hemoglobin, PNI: prognostic nutritional index.


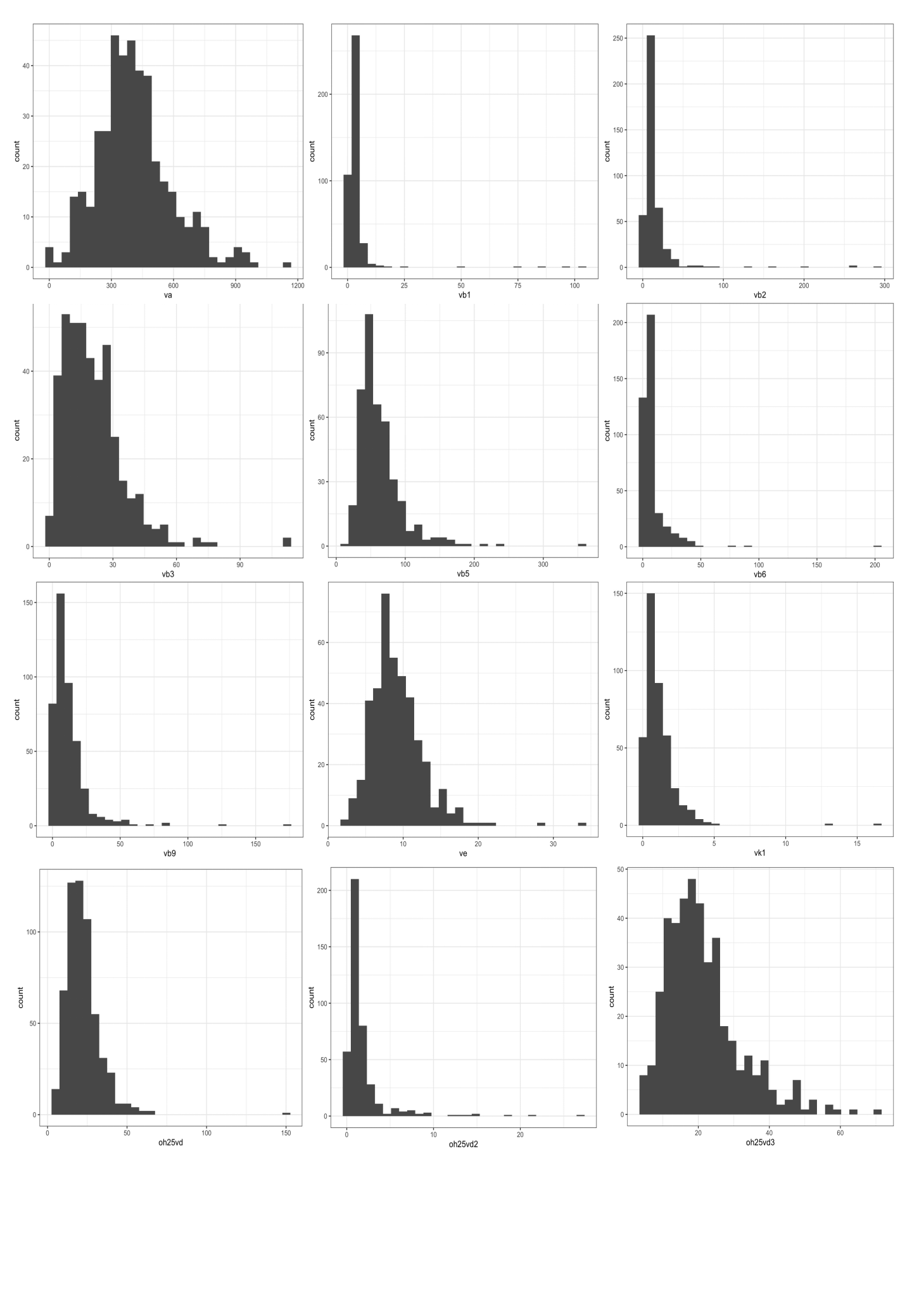


Supplementary Figure 1. The distribution plots of original vitamin levels. Figure A-J illustrates the original distribution of vitamins A, B1, B2, B3, B5, B6, B9, E, K1, 25-HO-D, 25-HO-D2, and 25-HO-D3.


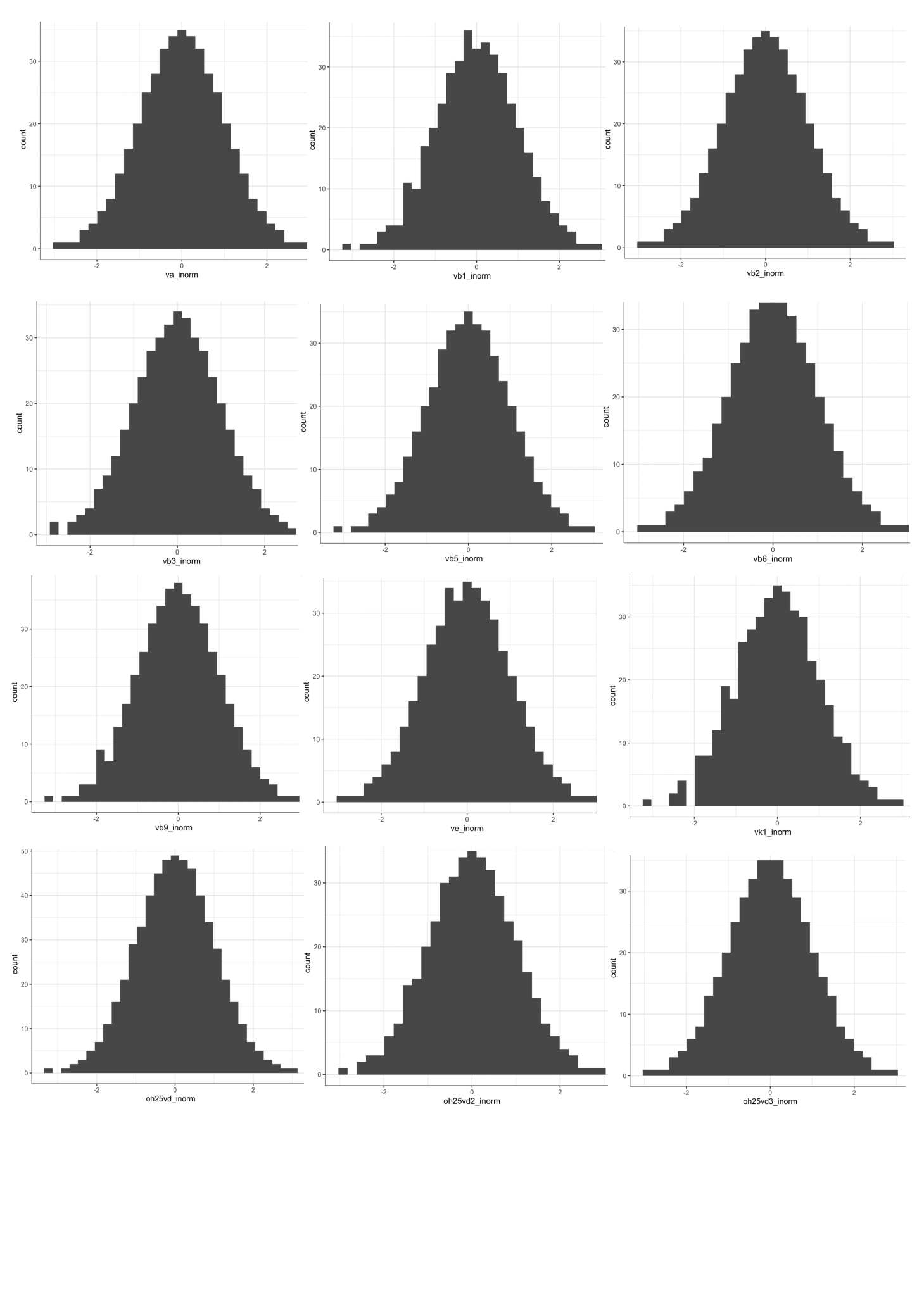


Supplementary Figure 2. The distribution plots of standardized vitamin levels. Figure A-J illustrates the standardized distribution of vitamins A, B1, B2, B3, B5, B6, B9, E, K1, 25-HO-D, 25-HO-D2, and 25-HO-D3.


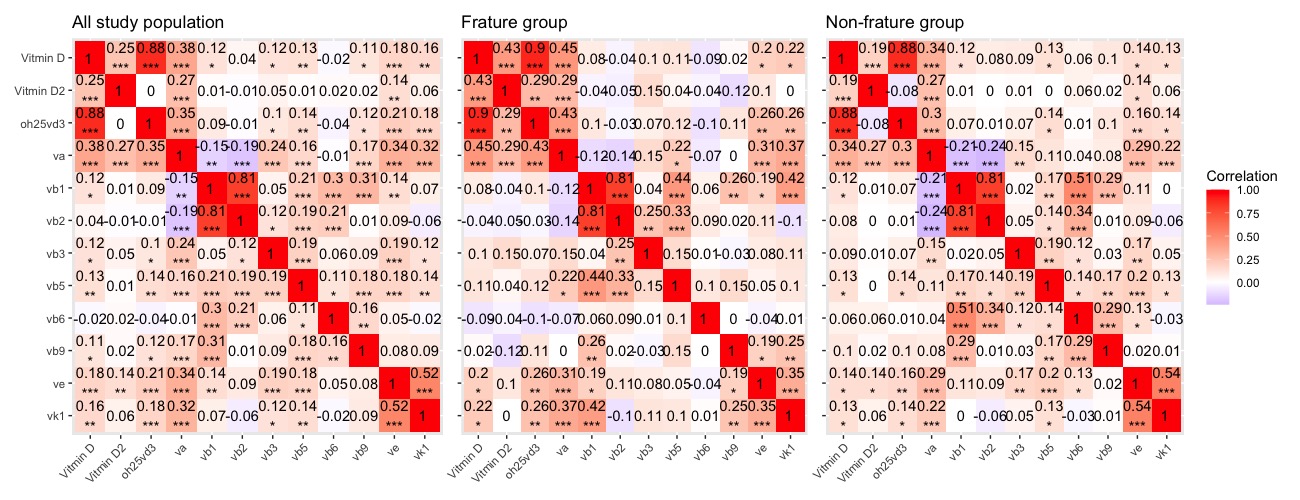


Supplementary Figure 3. Heatmap displaying the correlation matrix of various vitamins. Each cell represents the Pearson correlation coefficient between pairs of variables, with color intensity indicating the strength of the correlation. Red indicates a positive correlation, while blue indicates a negative correlation. Correlation values range from -1 to +1, with values closer to 1 or -1 denoting stronger relationships. P-values are indicated with asterisks as follows: * p < 0.05, ** p < 0.01, *** p < 0.001, highlighting the statistical significance of the correlations.
